# Stearoyl-CoA desaturase-1 promotes colorectal cancer metastasis in response to glucose by suppressing PTEN

**DOI:** 10.1186/s13046-018-0711-9

**Published:** 2018-03-12

**Authors:** Hui Ran, Yemin Zhu, Ruyuan Deng, Qi Zhang, Xisheng Liu, Ming Feng, Jie Zhong, Shuhai Lin, Xuemei Tong, Qing Su

**Affiliations:** 1 Department of Endocrinology, Xinhua Hospital, Shanghai Jiao Tong University School of Medicine, 1665, Kong Jiang Road, Shanghai, 200092 China; 2 Department of Biochemistry and Molecular Cell Biology, Shanghai Key Laboratory for Tumor Microenvironment and Inflammation, Key Laboratory of Cell Differentiation and Apoptosis of Chinese Ministry of Education, Shanghai Jiao Tong University School of Medicine, 280 S. Chongqing Road, Shanghai, 200025 China; 3 Department of General Surgery, Shanghai General Hospital, Shanghai Jiao Tong University School of Medicine, 100, Haining Road, Shanghai, 200080 China

**Keywords:** SCD1, PTEN, High glucose, Colorectal cancer, ChREBP, Metastasis

## Abstract

**Background:**

Diabetic patients have a higher risk factor for colorectal cancer (CRC) metastasis. Stearoyl-CoA desaturase 1 (SCD1), the main enzyme responsible for producing monounsaturated fatty acids(MUFA) from saturated fatty acids, is frequently deregulated in both diabetes and CRC. The function and mechanism of SCD1 in metastasis of CRC and its relevance to glucose remains largely unknown.

**Methods:**

SCD1 expression levels were analyzed in human CRC tissues and the Cancer Browser database (https://genome-cancer.ucsc.edu/). CRC cell lines stably transfected with SCD1 shRNAs or vector were established to investigate the role of SCD1 in modulating migration and invasion of CRC cells. A glucose concentration gradient was set to investigate regulation of SCD1 in CRC relevant to diabetic conditions.

**Results:**

The clinical data analysis showed high expression of SCD1 in CRC tissues with a negative correlation with the prognosis of CRC. In vitro experiments revealed that SCD1 increased CRC progression through promoting epithelial–mesenchymal transition (EMT). Lipidomic analysis demonstrated that SCD1 increased MUFA levels and MUFA administration could rescue migration and invasion defect of CRC cells induced by SCD1 knockdown. Furthermore, SCD1-mediated progression of CRC was promoted by carbohydrate response-element binding protein (ChREBP) in response to high glucose. Mechanistically, hyperglycemia-SCD1-MUFA induced CRC cell migration and invasion by regulating PTEN.

**Conclusions:**

Our findings show that SCD1 promotes metastasis of CRC cells through MUFA production and suppressing PTEN in response to glucose, which may be a novel mechanism for diabetes-induced CRC metastasis.

**Electronic supplementary material:**

The online version of this article (10.1186/s13046-018-0711-9) contains supplementary material, which is available to authorized users.

## Background

Colorectal cancer is one of the most common cancers worldwide, with rising incidence and mortality [1, 2]. Despite some significant improvements achieved in CRC diagnosis and treatment, the poor long-term follow-up still exists, which is caused by the CRC local recurrence and distant metastasis [3, 4]. The underlying mechanism of poor prognosis of CRC remains unclear.

Cancers have been considered as metabolic disorders, including high glycolysis, increased glutamine consumption and abnormal lipid metabolism [5–7]. One of the hallmarks of cancer cells is deregulation of lipid metabolism [8, 9]. Stearoyl-CoA desaturase-1, the fatty acyl Δ9-desaturing enzyme that converts saturated fatty acids (SFA) to monounsaturated fatty acids (MUFA) and is regulated by sterol regulatory element binding transcription factor 1 c (SREBP1c) or carbohydrate response-element binding protein, has been positively associated with many human malignancies [10–12]. Fatty acid biosynthetic pathway and SCD1 have been implicated to be essential for tumor cell survival [13, 14]. High expression of SCD1 and disorders of MUFA are involved in progression of cancers including hepatocellular, lung, renal, colorectal and prostate cancer [15–19]. SCD1 expression increased in erythrocytes of patients with CRC [20], but the relationship between SCD1 expression and CRC progression remains to be elucidated.

SCD1 has also been positively associated with insulin resistance and diabetes [21–23]. SCD1 activity has been suggested to be a risk factor for diabetes in humans [24, 25]. Increasing epidemiological studies showed positive correlation between type 2 diabetes mellitus (T2DM) and increased incidence and mortality of many cancers [26–29]. CRC remains as one of the most common diabetes-related cancers [27, 30, 31]. Although diabetic conditions have been associated with invasion and metastasis of CRC [29], the molecular mechanism underlying this connection remains elusive.

PTEN, a classical tumor suppressor gene, is the most important negative regulator of the PI3K/Akt signaling pathway [32–34]. PTEN function is commonly lost in a large proportion of human cancers, such as brain, breast, prostate and colorectal cancer, through somatic mutations, gene silencing, or epigenetic mechanisms [32, 35]. Studies have showed that loss of PTEN function contributes to progression of CRC [35].

In order to elucidate the role of SCD1 in CRC metastasis, we first analyzed the level of SCD1 in CRC tissues and found a negative correlation between SCD1 and the prognosis of CRC. Next, we showed that SCD1 promoted CRC progression through increasing MUFA levels and suppressing PTEN. Moreover, SCD1-induced cell migration and invasion contributed to glucose-induced CRC metastasis. These results provide us with novel insights into metastasis of CRC, especially in CRC patients with diabetes.

## Methods

### Clinical tissue specimens and database analysis

Pairs of cancer and adjacent noncancerous tissues enrolled in this study were collected from patients diagnosed with colorectal cancer, which obtained from Shanghai General Hospital of Shanghai Jiao Tong University. The study protocol was approved by and in accordance with the guidance of the Research Ethics Committee of Shanghai Jiao Tong University of Medicine, Shanghai, China. The Cancer Browser was linked from the UCSC Cancer Genomics Browser (https://genome-cancer.ucsc.edu/), colorectal cancer was chosen and then downloaded related datasets. SCD1 items were searched and summed up and then we combined SCD1 expression with clinical information, to build overall survival curve and outcomes comparison.

### Cell lines and culture conditions

Human colonic carcinoma cell lines HCT116, Caco2, HT29, SW116 and SW620 were cultured in Dulbecco’s Modified Eagle’s Medium (DMEM) (Giboco, USA), which contained 10% FBS, 2 mM L-glutamine and 1% penicillin/streptomycin (Gibco, USA). Cells were incubated in 37 °C, 5% CO2 humidified atmosphere.

### Quantitative real-time PCR

Total RNA was isolated by using TRIzol (Invitrogen Life Technologies, USA). cDNA was made using the PrimeScript™ RT reagent kit (Takara Bio Inc., Japan). Real Time-PCR was performed in triplicate utilizing StepOnePlus™ Real-Time PCR System (Applied Biosystems, USA). The primers were as follows: SCD1 forward, 5′- GTCCTTATGACAAGAACATTAGCC -3′ and reverse, 5′- AATCAATGAAGAATGTGGTGAAG -3′; ChREBP forward, 5′- GTGTCTCCCAAGTGGAAGAATTT -3′ and reverse, 5′- GCTCTTCCTCCGCTTCACAT -3′; 18 s rRNA forward, 5′- GTAACCCGTTGAACCCCATT-3′′ and reverse, 5′- CCATCCAATCGGTAGTAGCG-3′; The relative gene expression levels were calculated by the 2-ΔΔCt method. 18 s rRNA were used as an internal control.

### Western blotting

The protein lysates of cells or tissue specimens were prepared with RIPA buffer containing protease inhibitors (Thermo Fisher Scientific, USA) and supernatant was collected. Lysates were separated by 8.5% SDS-PAGE, and transferred to PVDF membranes (Millipore). Membranes were probed with the antibodies: anti-SCD1 (Proteintech Group, USA), anti-ChREBP (Santa Cruze, USA), anti-PTEN (Proteintech Group, USA), anti-Ecadherin (Cell Signaling Technology, USA), anti-Vimentin (Cell Signaling Technology, USA), anti-Akt (Cell Signaling Technology, USA); anti-phospho-Akt (Cell Signaling Technology, USA),anti-β-Actin (Cell Signaling Technology, USA), and anti-α-Tubulin(Santa Cruz Bio-technology, USA), followed by incubation of secondary peroxidase-labeled antibody (Cell Signaling Technology, USA). Protein signals were revealed with ECL detection system.

### Immunohistochemical staining

Harvested tumors were fixed in 4% paraformaldehyde, dehydrated, and embedded in paraffin. After deparaffinization and rehydration, the slides were retrieved for 30 min at 95 °C. 10% normal goat serum were used as blocking solution for 1 h. Then sections were subjected to antibody incubation overnight at 4 °C, followed by incubation with secondary antibody incubation. Then chromogenic reaction was performed with 3, 3-diaminobenzidine (Sigma-Aldrich, USA). After counterstaining with hematoxylin, slides were evaluated under a light microscope.

### Stable gene transfection

Short hairpin RNA (shRNA) against SCD1 (shSCD1) and nonspecific control shRNA were synthesized (Genepharma, China). The sequences of the two designed SCD1 shRNAs were as follows: shSCD1–1, CAGGACGATATCTCTAGCTCC; shSCD1–2, CCTACCTGCAAGTTCTACACC. The cDNA encoding human SCD1 was obtained from Dr. Jiahuai Han (Xiamen, China) and subcloned into vector pLVX-IRES-ZsGreen1 (Clontech). Cells were transfected with SCD1 shRNA and cDNA using polybrene as previous study [36].

### Transient gene transfection

Control and PTEN siRNA were synthesized by GenePharma Co., Ltd. (Shanghai, China). The siRNA sequences were as follows, si1 forward, 5′- GGCUAAGUGAAGAUGACAATT -3′ and reverse, 5′- UUGUCAUCUUCACUUAGCCTT -3′ and si2 forward, 5′- GAAGGCGUAUACAGGAACATT -3′ and reverse, 5′- UGUUCCUGUAUACGCCUUCTT -3′. The siRNA transfection was carried out using Lipofectamine RNAiMAX (ThermoFisher, USA), according to manufacturer’s protocol. The overexpression human PTEN was constructed by pEX-3(pGCMV/MCS/Neo) vector.

### Migration and invasion assay

24-Well transwell chambers (8 μm pore size; Becton Dickison, Falcon, USA) were used to measure the cell migration and invasion capacity. Approximately 5 × 10^5^ CRC cells in serum-free DMEM were added into the upper chambers with (invasion assays) or without (migration assays) matrigel coating, while complete DMEM was placed in the bottom. After 24 h, cells migrating or invading the bottom layers were fixed with 4% paraformaldehyde, following by staining with 0.1% crystal violet. The nonmigratory cells on the upper side of the membrane were scraped off with a cotton tip. Migrated and invasive cells were photographed under an inverted microscope and counted using the ImageJ software.

### Animals

BALB/c nude mice and C57BL/6 mice were originally provided by Shanghai Slack in Laboratory Animal Ltd. The animals were maintained under a specific pathogen free (SPF) condition with 12/12 h light/dark cycles. All animal experiments were approved by the Shanghai Jiao Tong University School of Medicine Institutional Animal Care and Use Committee (IACUC).

Male BALB/c nude mice (6 weeks old) were used for tumor metastasis study. Briefly, 2 × 10^6^ HCT116 cells stably transfected with shNC or shSCD1 were suspended in 100 μl PBS and injected through tail vein. Mice were sacrificed 6 weeks later, and lungs were harvested for histological examination of metastasis.

Male mice at 8 weeks of age were used for insulin resistance/T2DM model. Mice were maintained in 12/12 h light/dark cycles and fed either chow diet or high-fat diet (D12492, Research Diets). After 3 months, IPGTT and ITT were performed and mice were sacrificed.

### Lipidomics

The shNC and shSCD1 HCT116 cells were harvested when cell confluence was over 90%. After a series of addition of cold methanol/water (4:3, *v*/v), chloroform and centrifugation, the chloroform layers were collected and dried under gentle nitrogen stream. For the lipid analysis, lipid extract was reconstituted in 150 μL isopropyl/alcohol/acetonitrile/water (2:1:1). After centrifuging at 14000 rpm for 10 min, the supernatants were injected. The lipid supernatants were analyzed via ultra-performance liquid chromatography (UPLC) coupled to a SYNAPT G2 HDMS time of flight-mass spectrometry (UPLC-TOF/MS) (Waters Corporation, Milford, MA) through an electrospray ionization positive and negative full scan mode. The scan range was m/z 150–1200 (for positive mode) and m/z 90–1000(for negative mode). Lipids were separated on a Waters Acquity UPLC HSS T3 column (1.8 μm, 100 mm*2.1 mm) equipped with a Waters Acquity UPLC HSS T3 VanGuard Pre-column (1.8 μm, 5 mm*2.1 mm) maintained at 55 °C. Gradient elution was adopted with a flow rate at 0.3 mL/min. The lipid data was analyzed using Progenesis QI software (Waters Corporation, Milford, MA).Abundance of lipids were normalized by cell counts.

After summarizing the linked fatty acid compositions in lipidomic data, we calculated the *p* value and log2 (fold change) and made the volcano plot by R-Studio, taking log2 (fold change) as X axis and –log10 (*P* value) as Y axis.

### Statistical analysis

All experiments were performed in triplicate. All data were present as mean ± standard deviation. All graphing and statistical analyses were performed using GraphPad Prism 6 software (GraphPad Software, La Jolla, CA, USA) and SPSS 19 (IBM SPSS, IBM, Armonk, NY, USA). Correlations between the level of SCD1 in CRC tissues and clinic-pathological parameters were analyzed by Fisher’s exact tests. Comparison of survival between groups was performed using the log-rank test and Kaplan-Meier curves were plotted. The other data statistics were performed with student’s *t*-test in two groups. *P* value < 0.05(*), *P* value < 0.01(**) and *P* value < 0.001(***) were set as statistical significance.

## Results

### SCD1 is highly expressed in CRC tissues and has a negative correlation with the prognosis of CRC

**Fig. 1:**
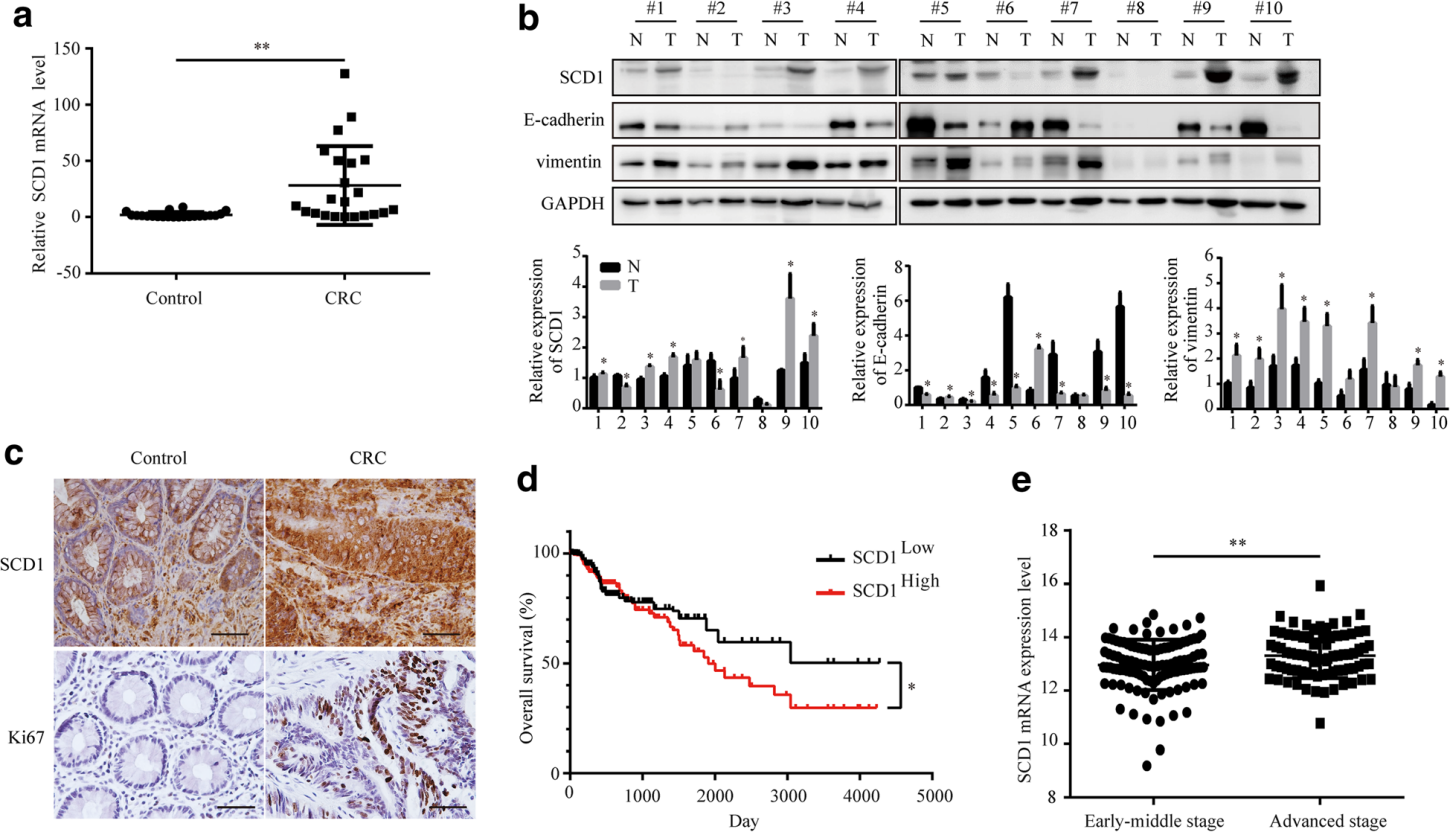
SCD1 is upregulated in human CRC tissues and associated with CRC prognosis. **a** SCD1 mRNA level in colorectal cancer tissues (CRC) and matched adjacent non-tumor tissues (Control) detected by Real Time-PCR. **b** Representative Western blot and quantification data of SCD1 and EMT markers (E-cadherin and vimentin) in colorectal cancer tissue and matched adjacent non-tumor tissue. **c** Representative images of immunohistochemmical staining of SCD1 and Ki67 in human cancer tissues and normal tissues (400×). The scale bar is 50 μm. **d** The overall survival (OS) rate of CRC patients with different SCD1 expression levels. **e** The relevance of SCD1 expression level to cancer stages of CRC patients

To determine whether SCD1 might play a role in CRC progression, we examined expression of SCD1 in cancer and adjacent normal samples of pre-treatment patients. The relative expression of SCD1 in CRC tissues was higher when compared with adjacent non-tumor tissues (Fig. 1a-c). SCD1 mRNA expression was significantly higher in 86.36% of CRC tissue samples (19/22), compared with adjacent non-tumor tissue (*P* = 0.002) (Fig. 1a). Additionally, western blotting analysis and immunohistochemical staining showed significantly increased SCD1 expression in CRC tissues, accompanied with higher expression of Ki67, a proliferation marker (Fig. 1b-c). We found that E-cadherin and vimentin, two proteins frequently altered during epithelial–mesenchymal transition (EMT) [37], was decreased and increased respectively in human colorectal cancer tissues, compared with the adjacent normal tissues (Fig. 1c).

Furthermore, the correlation between SCD1 expression and clinicopathological features of CRC patients was investigated. There was a positive correlation between SCD1 levels and lymph metastasis (*P* = 0.056) or tumor-node-metastasis (TNM) stages (*P* = 0.023, Table 1). To assess whether SCD1 might be involved in prognosis of CRC patients, relevance analysis of SCD1 expression with clinical pathological parameters from Cancer Browser was performed (https://genome-cancer.ucsc.edu/). Compared with patients with low SCD1 expression, CRC patients with high SCD1 levels have a poor overall survival (OS) (*P* = 0.043) (Fig. 1d). Furthermore, similar to the above clinicopathological features, high levels of SCD1 mRNA were associated with advanced TNM stages of CRC patients (*P* = 0.008) (Fig. 1e). These data indicated that SCD1 might play an important role in CRC progression.

**Table 1:** Association between SCD1 expression and clinicopathological characteristics of colorectal cancer patients

Clinical characteristics		SCD1 expression		
	Number of case	High (%)	Low (%)	*P*-value
Gender				
Male	16	12 (75)	4 (25)	1.00
Female	6	4 (66.67)	2 (33.33)	
Age(years)				
≥ 60	18	13 (72.22)	5 (27.78)	1.000
≤ 60	4	3 (75)	1 (25)	
Tumor size (cm^2^)				
<30	19	14 (73.68)	5(26.32)	1.000
≥ 30	3	2 (66.67)	1 (33.33)	
Differentiation				
Well	5	2 (40)	3 (60)	0.100
Moderate to poor	17	14 (82.35)	3 (17.65)	
Tumor location				
Colon	13	10 (76.92)	3 (23.08)	0.655
Rectum	9	6 (66.67)	3 (33.33)	
Lymph metastasis				
Absent	10	5 (50)	5 (50)	0.056
Present	12	11(91.67)	1 (8.33)	
Distant metastasis				
Absent	20	14 (70)	6 (30)	1.000
Present	2	2 (100)	0 (0)	
TNM stage				
I + II	9	4 (44.44)	5 (55.56)	0.023^*^
III + IV	13	12 (92.31)	1 (7.69)	

Samples with upregulated SCD1 expression showed a significant association with TNM stage (*P* = 0.023). One asterisk (*) indicated the statistical difference of *P* < 0.05

### SCD1 increases migration and invasion of colorectal cancer cells by promoting epithelial–mesenchymal transition

**Fig. 2:**
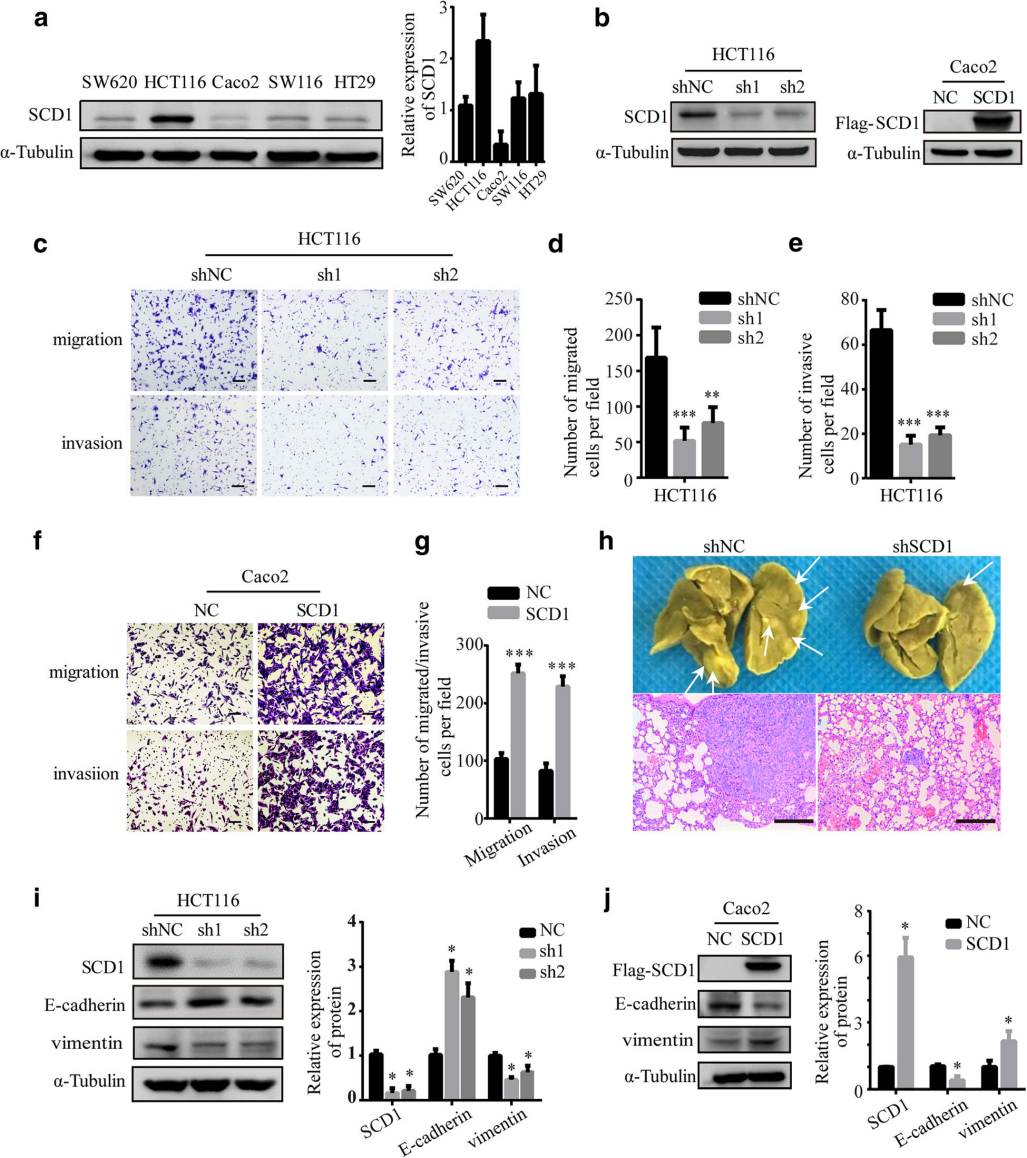
SCD1 promotes migration and invasion of colorectal cancer cells by regulating EMT. **a** Representative Western blot of SCD1 and quantification result in five colorectal cancer cell lines. **b** Protein levels of SCD1 in HCT116 cells transfected with shRNAs for SCD1 (sh1 and sh2) or Caco2 cells ectopically expressing SCD1. **c** Transwell assay of HCT116 cells after SCD1 knockdown. The scale bar is 100 μm. **d**, **e** Histograms show the numbers of migrated (**d**) and invasive (**e**) HCT116 cells. **f** Transwell assay of Caco2 cell lines after SCD1 overexpression. The scale bar is 100 μm. **g** Histograms show the numbers of migrated and invasive Caco2 cells. **h** Morphological analysis of lung metastasis in mice injected with HCT116 cells transfected with control shRNA or SCD1 shRNA (*n* = 8 per group). Arrows indicate metastatic nodules in the lungs. The scale bar is 200 μm. **i**, **j** Protein levels of E-cadherin and vimentin in colorectal cancer cells transfected with SCD1 shRNA (**i**) or SCD1 cDNA (**j**)

Since SCD1 had a positive correlation with CRC progression, we hypothesized that increasing SCD1 expression might accelerate CRC migration and invasion. To choose suitable CRC cell lines for further experiments, we examined the expression profile of SCD1 in five different human CRC cell lines including SW620, HCT116, Caco2, SW116 and HT29 by western blotting. The expression levels of SCD1 were higher in HCT116 and minimal in CaCo2. Moreover, the protein showed intermediate expression levels in SW620, SW116 and HT29 (Fig. 2a). In order to investigate the effect of suppressing SCD1 on metastatic ability of CRC cells, we designed two different SCD1 shRNAs (shSCD1) to stably knockdown SCD1 expression in HCT116 and SW116 cells (Fig. 2b, Additional file 1: Figure S1A). We found that SCD1 knockdown impaired the migration and invasion ability of CRC cells (Fig. 2c, Additional file 1: Figure S1B). Quantification results revealed a significant decrease in the number of migrated and invaded cells after SCD1 knockdown (Fig. 2d-e, Additional file 1: Figure. S1C-D). We also ectopically expressed SCD1 in Caco2 cells (SCD1) to study how increased SCD1 expression affected metastasis (Fig. 2b). Interestingly, SCD1 overexpression significantly increased migration and invasion rates of Caco2 cells (Fig. 2f-g). All of the results suggest that SCD1 promote migration and invasion of colorectal cancer cells.

To investigate the effect of SCD1 suppression on the metastatic potential of CRC cells in vivo, we employed a mouse tail vein lung metastasis model as described [38]. We found that the incidence of lung metastasis decreased in mice injected with HCT116-shSCD1 cells compared to those injected with HCT116-shNC cells. Histological analysis confirmed that suppression of SCD1 decreased the size and number of lung metastatic tumors (Fig. 2h).

Since SCD1 and EMT markers were simultaneously highly expressed in CRC specimens, we next investigated whether SCD1 could promote metastasis of CRC cells by modulating EMT. Inhibition of SCD1 resulted in significantly elevated expression of E-cadherin and decreased expression of vimentin in HCT116 cells (Fig. 2i). On the other hand, overexpression of SCD1 led to significantly decreased expression of E-cadherin and increased expression of vimentin (Fig. 2j). These data indicate that SCD1 can promote metastasis of CRC by regulating EMT.

### SCD1 enhances the migration and invasion ability of CRC cells by regulating fatty acids composition and increasing MUFAs levels

**Fig. 3:**
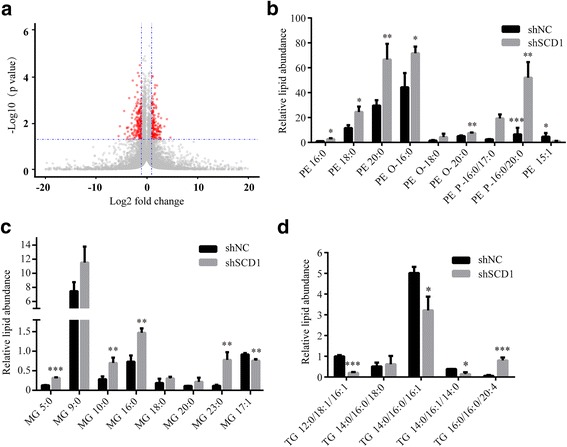
Lipidomics analysis for HCT116 cells transfected with shSCD1 or shNC. **a** Volcano plots show log2 fold-changes versus −log10 *P*-values of lipid species of HCT116 transfected with shSCD1 when compared with control cells. The horizontal line indicates the cut-off value for significance levels. Each dot represents a lipid species (red: lipid species *P* < 0.05 with a > 2 fold-change; grey: not significant). **b**-**d** Relative abundance of lipids extracted from shSCD1 or shNC-transfected HCT116 cells, including PE (**b**), MG (**c**) and TG (**d**)

As reported, SCD1 plays an important role in fatty acid desaturation, which influences membrane phospholipids unsaturation and signal transduction, contributing to the progression of many cancers [12, 14] . To assess the effect of SCD1 on lipid remodeling and fatty acid ratio, quantitative lipidomic analysis was conducted using extracts of shSCD1 (sh1) and shNC HCT116 cells. The effect of SCD1 knockdown were broad, changing many lipid species measured (Fig. 3a). Levels of some lipids, especially those consist of one or more MUFAs, were significantly reduced following SCD1 knockdown (Table 2). Significant increase in SFA levels and reduction in MUFA levels in phosphatidylethanolamine (PE), monoglyceride (MG) and triglyceride (TG), were observed in shSCD1 HCT116 cells (Fig. 3b-d). Overall, these results are consistent with a role of SCD1 in influencing the composition of lipids by changing MUFA/SFA ratio in CRC cells.

**Table 2:** Significantly different lipid species of shSCD1 and NC HCT116 cells

Sum composition	Molecular species composition		Relative abundance				
			shNC		shSCD1		
			Average	SD	Average	SD	*P*-Value
PC 34:2	**16:1/18:1**		1103.5873	18.1423	856.4450	24.2773	0.0162
PE 36:1	**P-18:0/18:1**		87.3496	11.9209	51.4006	32.5577	0.0003
PE 36:2	**16:1/20:1**	14:1/22:1	4.5314	1.2648	1.4709	1.2409	0.0101
PS 39:2	**17:1/22:1**	15:1/24:1	126.6432	39.8219	28.2973	26.8162	0.0046
PA 33:2	**15:1/18:1**	16:1/17:1	1.0000	0.1757	0.0529	0.0325	0.0009
PA 35:3	**17:2/18:1**	17:1/18:2	0.8327	0.1434	0.0503	0.0363	0.0010
PA 42:2	**18:1/24:1**	20:1/22:1	1.2632	0.0431	0.6991	0.2225	0.0125
MGDG 25:0	**16:0/9:0**	18:0/7:0	0.1367	0.0282	0.2856	0.1137	0.0200
MGDG 25:2	**17:1/18:1**	15:1/20:1	2.29352	0.2163	1.0015	0.2091	2.137E-05

Lipid species are annotated by sum composition and molecular species composition (bold indicates the most abundant molecular lipid species)

### SCD1 promotes CRC migration and invasion by increasing MUFA

**Fig. 4:**
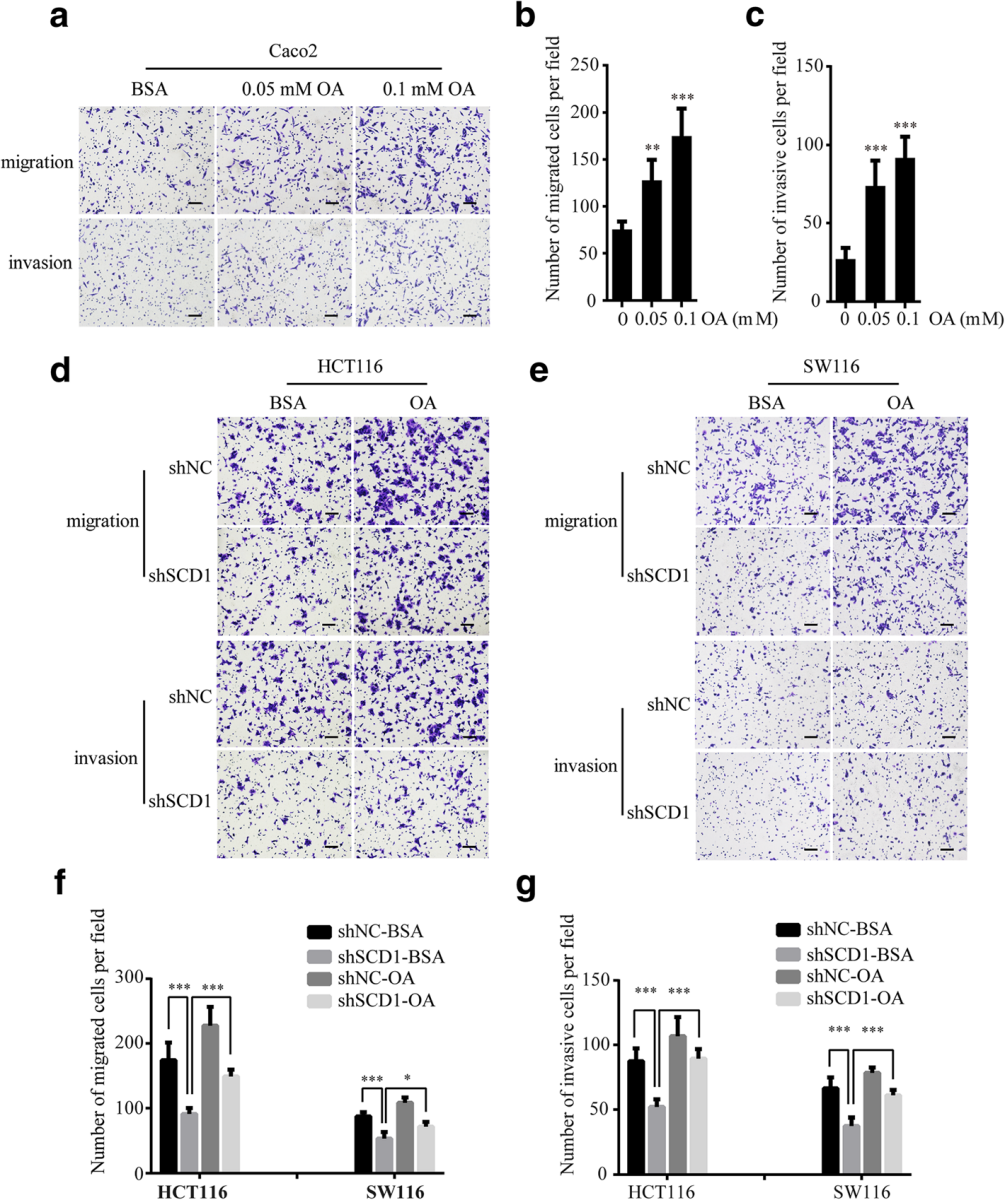
Effect of OA on migration and invasion ability of CRC cells. **a** Representative photographs of transwell assays of Caco2 cells after OA or BSA treatment for 48 h. The scale bar is 100 μm. **b**-**c** Histograms show the numbers of migrated (**b**) and invasive Caco2 cells (**c**). **d**-**e** Representative photographs of transwell assays of shSCD1 or shNC-transfected HCT116 (**d**) and SW116 (**e**) cells after 0.1 mM OA or BSA treatment. The scale bar is 100 μm. **f**-**g** Histograms show the numbers of migrated cells (**f**) and invasive cells (**g**)

Given the reduction of MUFA in shSCD1 CRC cells, we hypothesized that SCD1 could promote CRC progression through the effect of MUFA. Next, we examined the migration and invasion ability of CRC cells following treatment of oleic acid (OA, C18:1 n-9), the most common MUFA. To choose the best OA response condition, we treated Caco2 cells with different concentrations of OA (0, 0.05, 0.1 mM). After OA treatment, the migration and invasion rates of Caco2 cells were elevated, especially in response to 0.1 mM OA, which was similar to the effect of SCD1 overexpression (Fig. 4a-c). Interestingly, the migration and invasion ability reduced by SCD1 knockdown was reversed after OA treatment in HCT116 and SW116 cells (Fig. 4d-g). These results, together with the lipidomic profiling data, indicate that SCD1 accelerate colorectal cancer cell migration and invasion by increasing MUFA.

### High glucose promotes SCD1 expression and migration and invasion of colorectal cancer cells

**Fig. 5:**
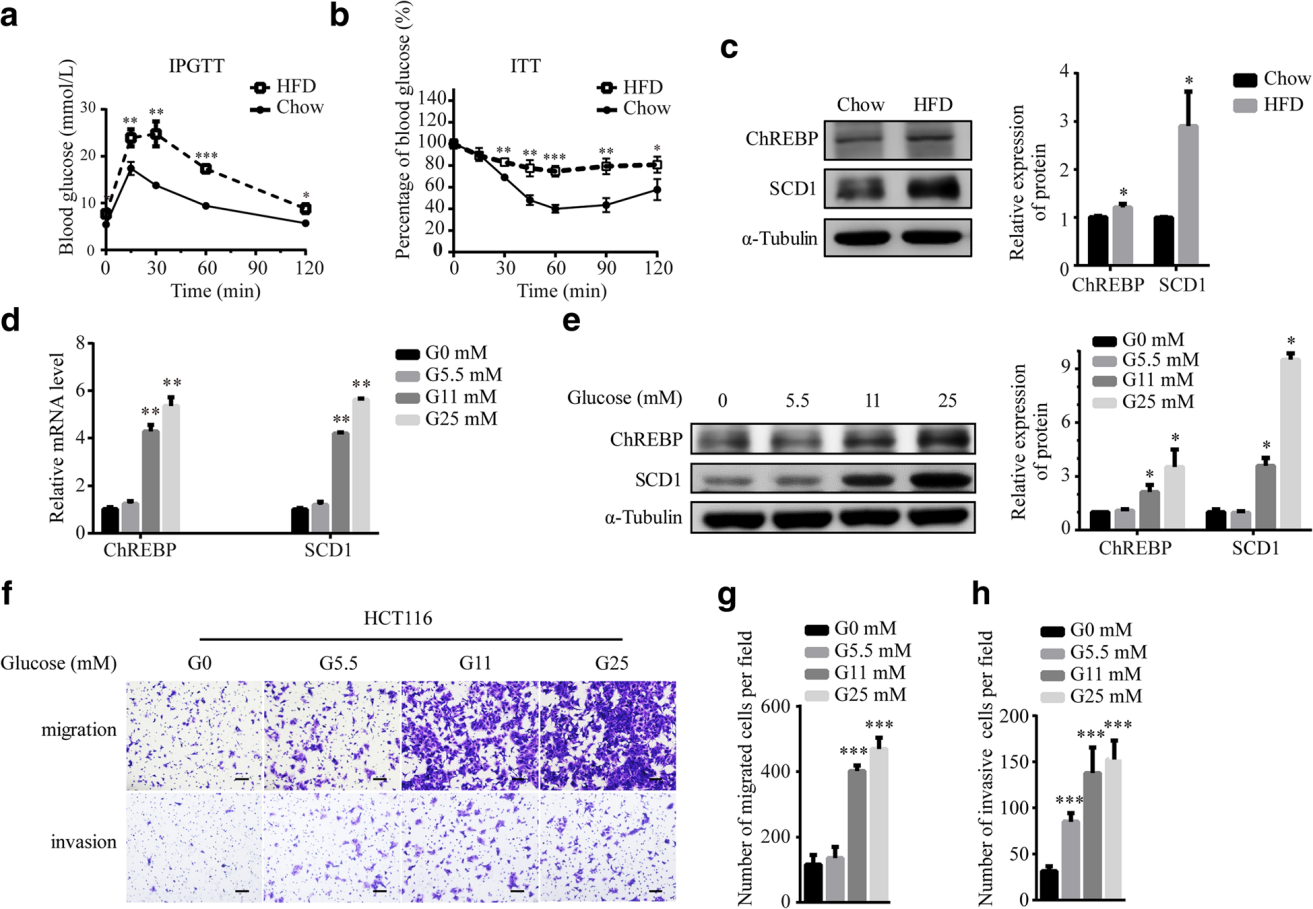
Hyperglycemia promotes SCD1 expression as well as migration and invasion of CRC cells. **a**, **b**) IPGTT (**a**) and ITT (**b**) of chow-fed and HFD-fed mice. **c** Representative Western blot and quantified results of SCD1 and ChREBP in colon tissues from chow and HFD fed mice. **d**, **e** HCT116 cells treated with glucose for 24 h and expression levels of SCD1 and ChREBP were determined by Real Time-PCR (**d**) and Western blot (**e**). **f** Transwell assay of HCT116 cells treated with 0 mM (G0), 5.5 mM (G5.5), 11 mM (G11) or 25 mM (G25) glucose. The scale bar is 100 μm. **g**, **h**) Histograms show the numbers of migrated cells (**g**) and invasive cells (**h**) under different glucose conditions

SCD1 gene expression is altered by nutrients such as glucose, insulin and lipids [39, 40]. To confirm whether T2DM has a positive connection with colorectal SCD1 expression, we conducted studies in C57BL/6 mice fed chow or high-fat-diet (HFD). Intraperitoneal glucose tolerance test and insulin tolerance test showed HFD group mice had hyperglycemia and insulin resistance (Fig. 5a-b). The protein level of SCD1 in colon of HFD-fed mice is higher than that of the chow-fed mice (Fig. 5c). Importantly, the transfactor, carbohydrate response-element binding protein (ChREBP), which can regulate SCD1 expression, showed increased expression level. The results suggest that T2DM promote expression of SCD1 in colon.

Glucose increases SCD1 transcription partly by activating ChREBP, a glucose-responsive transcription factor [11]. To test whether the original source of diabetic-enhanced SCD1 expression is hyperglycemia, we treated HCT116 cells with 0 mM (G0), 5.5 mM (G5.5), 11 mM (G11) and 25 mM glucose (G25). RT-PCR and western blotting results showed glucose treatment increased the expression of ChREBP and SCD1 in a concentration-dependent manner (Fig. 5d-e).

To determine whether hyperglycemia regulates metastasis of CRC, colorectal cancer cells were grown in the culture medium containing different amount of glucose. Compared with G0 and G5.5 groups, the migration and invasion ability of HCT116 and SW116 cells markedly increased in G11 and G25 groups (Fig. 5f-h, Additional file 2: Figure S2). The results suggest that high glucose accelerates colorectal cancer cells’ progression.

### SCD1 contributes to glucose-induced CRC migration and invasion

**Fig. 6:**
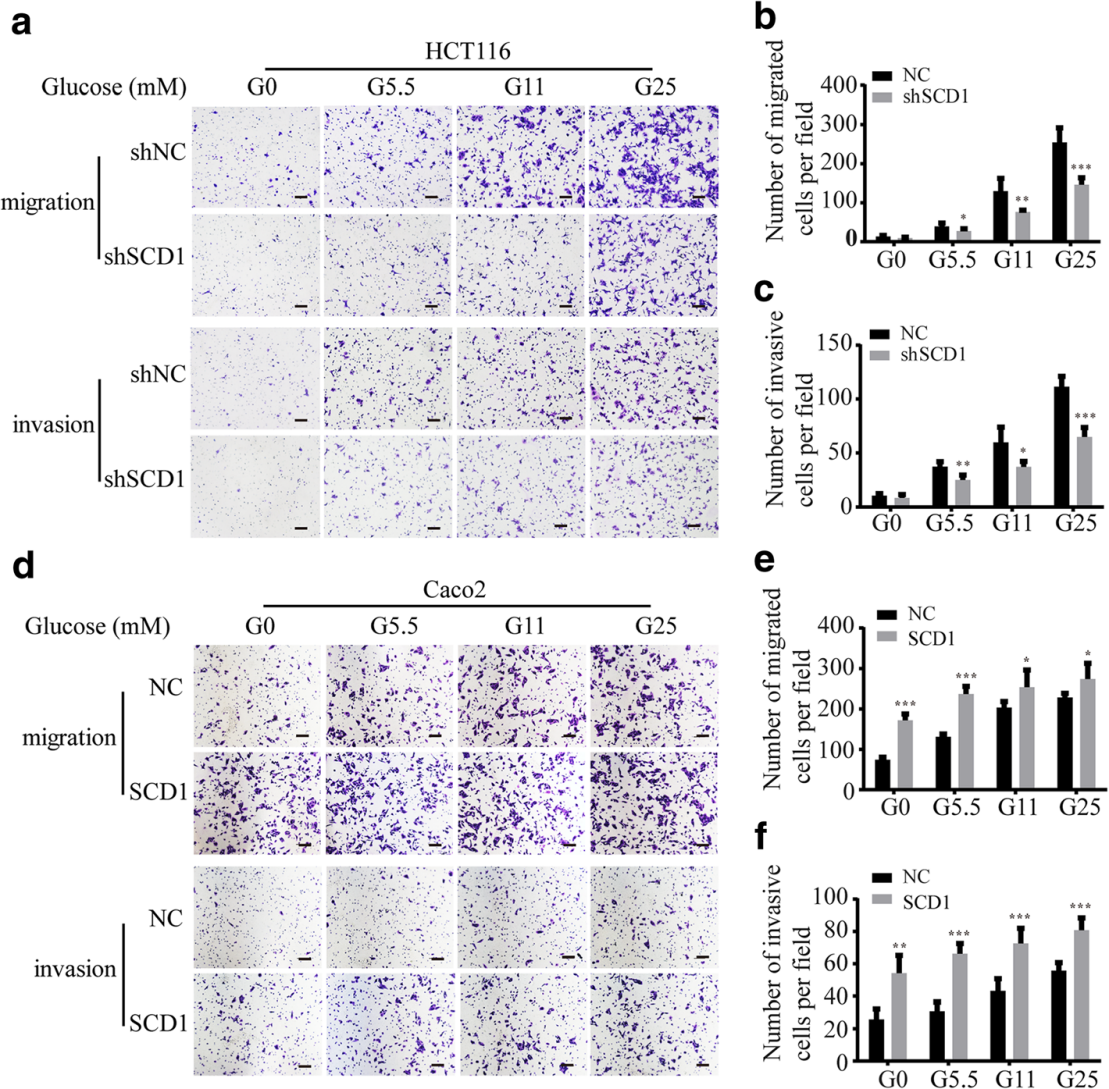
SCD1 contributes to glucose-induced migration and invasion of colorectal cancer cells. **a** Representative photographs of transwell assays of shSCD1 or shNC-transfected HCT116 cells after glucose treatment. The scale bar is 100 μm. **b**, **c**) Histograms show the numbers of migrated (**b**) and invasive (**c**) HCT116 cells. **d** Representative photographs of transwell assays of Caco2 cells ectopically expressing NC or SCD1 after glucose treatment. The scale bar is 100 μm. **e**, **f** Histograms show the numbers of migrated (**e**) and invasive Caco2 cells (**f**)

We next investigated whether SCD1 played an important role in glucose-induced CRC migration and invasion. We found that SCD1 knockdown impaired the migration and invasion ability of HCT116 and SW116 cells cultured under different glucose concentrations (0–25 mM) for 24 h (Fig. 6a-c, Additional file 3: Figure S3). Interestingly, compared to the shNC group, colorectal cancer cells transfected with shSCD1 showed more modest increase in migration and invasion in response to glucose (Fig. 6a-c, Additional file 3: Figure S3). Moreover, the enhanced migration and invasion rates of Caco2 cells ectopically expressing SCD1 showed just mild increase after glucose treatment (Fig. 6d-f). All of the results suggest that SCD1 contributes to glucose-induced CRC migration and invasion.

### SCD1-MUFA promotes migration and invasion of colorectal cancer cells by suppressing PTEN/Akt pathway

**Fig. 7:**
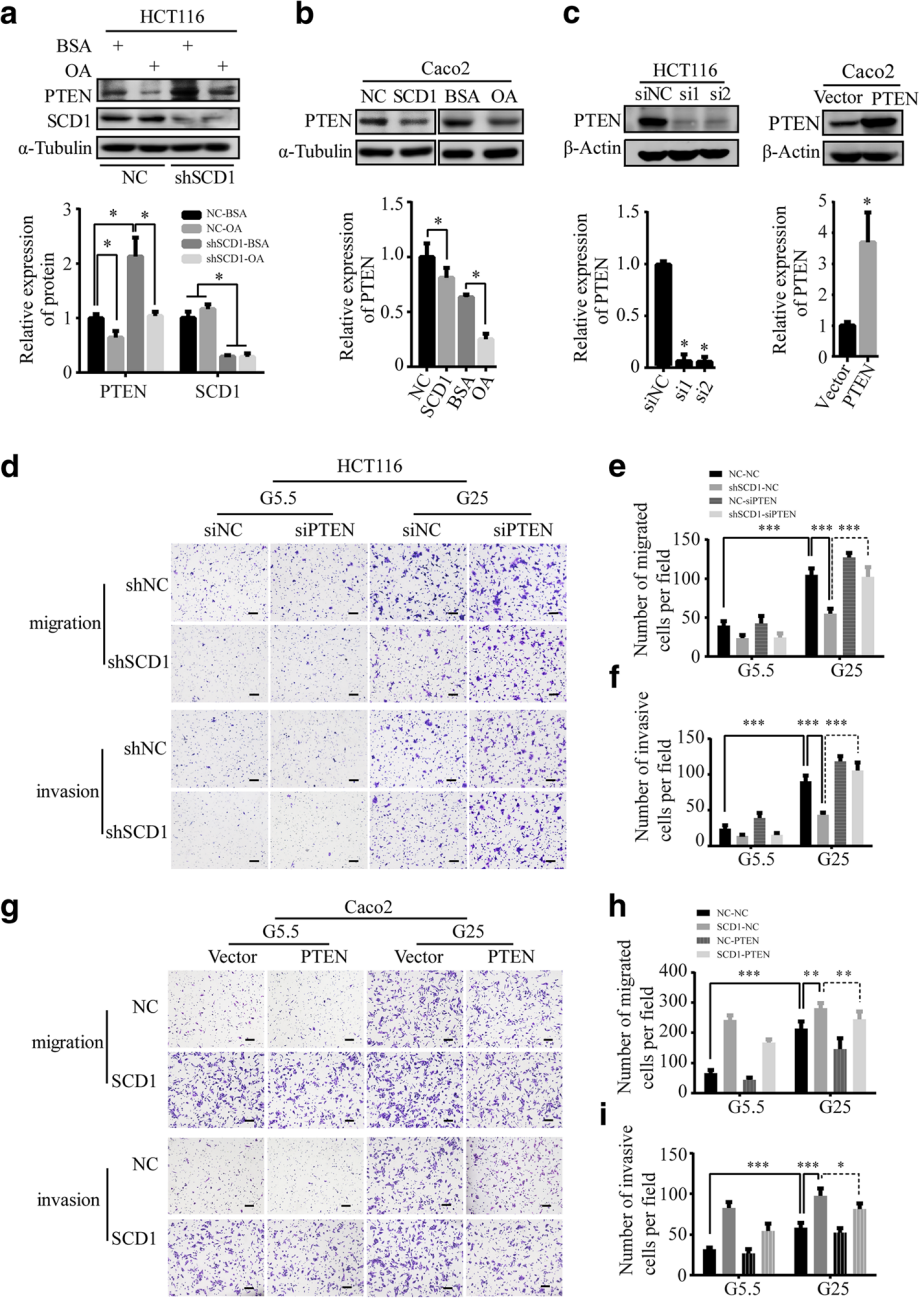
PTEN plays an important role in SCD1-induced migration and invasion of CRC cells. **a**, **b** Representative Western blot and quantification data of PTEN proteins of OA-treated HCT116 (**a**) and Caco2 (**b**) cells. **c** Representative Western blot and quantification data of PTEN in HCT116 cells transfected with siRNAs for PTEN (si1 and si2) or Caco2 cells ectopically expressing PTEN. **d** Representative photographs of transwell assays of shSCD1 or shNC-transfected HCT116 after being transfected with PTEN siRNAs (siPTEN) or negative control scramble siRNAs (siNC). The scale bar is 100 μm. **e**, **f** Histograms show the numbers of migrated (**e**) and invasive (**f**) HCT116 cells. **g** Transwell assay of SCD1 overexpression Caco2 cell lines after ectopically expressing PTEN. The scale bar is 100 μm. **h**, **i** Histograms show the number of migrated (**h**) and invasive (**i**) Caco2 cells

Several signaling pathways such as PTEN, Wnt/β-Catenin, STAT3, mTOR/S6K and JNK have been reported to play important roles in CRC metastasis [41–47]. We found that the activity of β-Catenin, STAT3, S6K and JNK was unchanged after SCD1 knockdown or overexpression in CRC cells (Additional file 4: Figure S4A). Notably, SCD1 knockdown increased the expression of PTEN and OA treatment could reverse the change in HCT116 cells (Fig. 7a). Conversely, SCD1 overexpression or treatment of OA, the common SCD1 product, significantly decreased the level of PTEN in Caco2 cells (Fig. 7b). Our findings agreed with previous findings of unsaturated fatty acids downregulating PTEN in liver cancer [41, 42]. These data suggest that SCD1-MUFA might act as a negative regulator for PTEN and induce progression of CRC.

We next examined whether upregulation of PTEN was responsible for changes in CRC cell migration and invasion after SCD1 knockdown (Fig. 7c, Additional file 4: Figure S4B). Compared with the G5.5 group, the G25 group showed increased migration and invasion rate (Fig. 7d-f, Additional file 4: Figure S4C-E). Importantly, PTEN knockdown (siPTEN) reduced shSCD1-induced downregulation in migration and invasion of CRC cells, especially in the high glucose condition (Fig. 7d-f, Additional file 4: Figure S4C-E). Conversely, ectopic PTEN expression reduced SCD1-induced and glucose-induced migration and invasion of CRC cells (Fig. 7c, g-i). In HCT116 cells transfected with shSCD1, PTEN was upregulated and phosphate-Akt was reduced. On the other hand, the change of E-cadherin and vimentin expression level induced by high glucose was weakened after SCD1 knockdown (Additional file 4: Figure S4F).

Taken together, our results indicate that SCD1, via MUFA, suppress the expression of PTEN and activity of Akt, promoting the migration and invasion of colorectal cancer cells.

## Discussion

CRC is one of the most common malignancies which have a higher morbidity in diabetic patients [2]. Upregulation of SCD1 activity and/or expression has been reported as a risk factor for CRC [18, 48]. Besides, after being treated with SCD1 antagonist, the growth of xenograft tumors generated from HCT116 cells was reduced in mice [49]. However, the mechanistic role of SCD1 in CRC metastasis remains to be elucidated. Poor prognosis of CRC is usually due to tumor metastasis and recurrence. So, identification of the role of SCD1 in CRC progression will provide effective strategies to improve advanced CRC patients’ prognosis.

Accumulating data indicate elevated SCD1 activity and increased levels of MUFAs in parallel to reduced SFA levels present in neoplastic cells and tumor tissues [14, 50]. In our study, analysis of human CRC tissues and CRC database revealed high SCD1 levels in human CRC tissues and SCD1 had a negative correlation with CRC prognosis. Moreover, our in vitro study showed SCD1 could promote migration and invasion of CRC cells by regulating EMT. The lipidomics result in this study showed that MUFA levels and the ratio of MUFA and SFA in lipids of CRC cells were reduced after SCD1 knockdown, leading to a difference in the relative abundance of many lipid fractions. Furthermore, we found that MUFA stimulated migration and invasion of CRC cells and could reverse the reduced migration and invasion rates of SCD1 knockdown cells, which is consistent with the notion that high SCD1 levels and subsequently altered fatty acid composition are biochemical features of cancers [14].

T2DM, a common metabolic disorder, often results in hyperglycemia, insulin resistance and dyslipidemia. As one of the target genes of ChREBP, a glucose sensing transcription factor, SCD1 plays an important role in T2DM [51]. Our previous study showed advanced glycation end products (AGEs) in T2DM promoted CRC cell proliferation by increasing ChREBP expression [52]. Here, our data demonstrated that glucose increased expression of both ChREBP and SCD1 in CRC cells. Taken together, these results suggested the high glucose could induce upregulation of colorectal SCD1 and this may be mediated by activation of ChREBP.

Previous meta-analysis and pathologic analysis showed CRC patients with diabetes have more tumor invasion, higher TNM staging and increased mortality, compared to those without diabetes [53, 54]. Our previous study showed diabetes aggravates CRC by increasing specificity protein 1 (Sp1) expression [55]. However, whether SCD1 is induced in the process of diabetes-related CRC progression is elusive. In this study, we used CRC cell lines to verify high glucose promoted CRC cells migration and invasion. These data are consistent with epidemiological studies that diabetic CRC patients have a poor outcome and higher mortality [54]. In addition, the promotion of migration and invasion by high glucose showed positive correlation with the level of SCD1. Our results indicate that SCD1 may be part of the diabetic CRC development mechanism.

As mentioned above, high SCD1 activity and elevated unsaturated fatty acids levels are common signs of cancers. Several previous studies suggest that unsaturated fatty acids can promote hepatoma and hepatic steatosis progression through downregulation of PTEN [41, 42, 56]. Our data showed high glucose increased expression of SCD1, leading to MUFA-induced CRC progression by suppressing PTEN and promoting EMT. The results were consistent with findings that loss of function of PTEN can promote cancers progression by regulating EMT in many cancers [57, 58]. Furthermore, after changing PTEN expression levels, the inhibitory or promoting effect caused by SCD1 knockdown or overexpression alleviated. Previous study showed that high glucose could promote cancer progression by suppressing PTEN in human breast cancer cells [59]. The relationship between hyperglycemia and EMT has been revealed in several cancers [58–61]. We observed that high glucose conditions led to decreased PTEN expression and increased EMT, accompanied by increased SCD1 expression in HCT116 cells, compared with physiological glucose concentrations. Consequently, protein levels of EMT markers and phosphorylation of AKT, which were under the regulation of PTEN, altered accordingly after SCD1 knockdown. Therefore, these data indicate that high glucose-induced CRC progression may be partly due to downregulation of PTEN and its downstream EMT.

## Conclusions

**Fig. 8:**
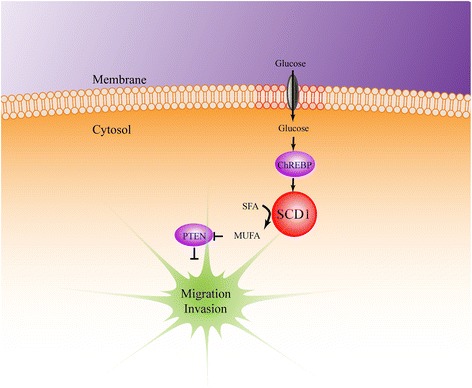
Schematic diagram describing the functional significance of SCD1 in glucose-induced progression of CRC. SCD1 contributes to glucose-induced migration and invasion of CRC by increasing MUFA production to inhibit PTEN

In conclusion, our findings have demonstrated that SCD1 acts as an important mediator in hyperglycemia-induced promotion of CRC cells migration and invasion. The relative contribution of SCD1 to glucose-induced CRC cell migration and invasion depends on MUFA suppressing PTEN/Akt signaling and regulating EMT (Fig. 8). Therefore, our findings provide a rationale for future studies aiming at validating this pathway as a potential therapeutic target for CRC.

## Additional files

Additional file 1: **Figure S1.** SCD1 promotes migration and invasion of SW116 cells. (A) Quantification results of SCD1 in HCT116 cells transfected with shRNAs for SCD1 (sh1 and sh2) or Caco2 cells ectopically expressing SCD1. (B) Representative Western blot and quantified results of SCD1 in SW116 cells transfected with shRNAs for SCD1 (sh1 and sh2). (C) Transwell assay of SW116 cells after SCD1 knockdown. The scale bar is 100 μm. (D, E) Histograms show the numbers of migrated (D) and invasive (E) SW116 cells. (TIFF 1383 kb)

Additional file 2: **Figure S2.** Glucose promotes migration and invasion of SW116 cells. (A) Transwell assay of SW116 cells treated with 0 mM (G0), 5.5 mM (G5.5), 11 mM (G11) or 25 mM (G25) glucose. The scale bar is 100 μm. (B, C) Histograms show the number of migrated (B) and invasive (C) SW116 cells. (TIFF 1154 kb)

Additional file 3: **Figure S3.** Effect of glucose on SCD1-induced migration and invasion ability of SW116 cells. (A) Representative photographs of transwell assays of shSCD1 or shNC-transfected SW116 cells after glucose treatment. The scale bar is 100 μm. (B, C) Histograms show the numbers of migrated (B) and invasive (C) SW116 cells. (TIFF 1277 kb)

Additional file 4: **Figure S4.** PTEN mediates SCD1-induced migration and invasion of SW116 cells. (A) Representative Western blot of SCD1, β-Catenin, STAT3, S6K and JNK in CRC cells transfected with shSCD1 or SCD1 cDNA. (B) Representative Western blot and quantification data of PTEN in SW116 cells transfected with siRNAs for PTEN (si1 and si2). (C) Representative photographs of transwell assays of shSCD1 or shNC-transfected SW116 after being transfected with PTEN siRNAs (siPTEN) or negative control scramble siRNAs (siNC). The scale bar is 100 μm. (D, E) Histograms show the numbers of migrated (D) and invasive (E) SW116 cells. (F) Representative Western blots and quantified results of SCD1, PTEN, Akt, p-Akt (Ser473), p-Akt (Thr308), E-cadherin and vimentin. (TIFF 2175 kb)

## Abbreviations

AGEs: advanced glycation end products
ChREBP: carbohydrate response-element binding protein
CRC: colorectal cancer
EMT: epithelial–mesenchymal transition
HFD: high-fat-diet
MUFA: monounsaturated fatty acids
OA: oleic acid
SCD1: Stearoyl-CoA desaturase-1
SFA: saturated fatty acids
Sp1: specificity protein 1
SREBP1c: sterol regulatory element binding transcription factor 1 c
T2DM: type 2 diabetes mellitus
