# Correction: Stearoyl-CoA desaturase-1 promotes colorectal cancer metastasis in response to glucose by suppressing PTEN

**DOI:** 10.1186/s13046-025-03300-2

**Published:** 2025-02-01

**Authors:** Hui Ran, Yemin Zhu, Ruyuan Deng, Qi Zhang, Xisheng Liu, Ming Feng, Jie Zhong, Shuhai Lin, Xuemei Tong, Qing Su

**Affiliations:** 1 Department of Endocrinology, Xinhua Hospital, Shanghai Jiao Tong University School of Medicine, 1665, Kong Jiang Road, Shanghai, 200092 China; 2 Department of Biochemistry and Molecular Cell Biology, Shanghai Key Laboratory for Tumor Microenvironment and Inflammation, Key Laboratory of Cell Differentiation and Apoptosis of Chinese Ministry of Education, Shanghai Jiao Tong University School of Medicine, 280 S. Chongqing Road, Shanghai, 200025 China; 3 Department of General Surgery, Shanghai General Hospital, Shanghai Jiao Tong University School of Medicine, 100, Haining Road, Shanghai, 200080 China

**Correction** **:** **J Exp Clin Cancer Res 37, 54 (2018)**

**https://doi.org/10.1186/s13046-018-0711-9**

Following the publication of the original article [1], the authors identified errors in Figure 7G and Supplementary Material Figure S3.

- Figures 7G – Invasion result of NC-NC(G25)
- Figure S3 – Invasion result of shNC(G25)

The correct figures are given below.

**Incorrect Fig. 7**

**Fig. 7:**
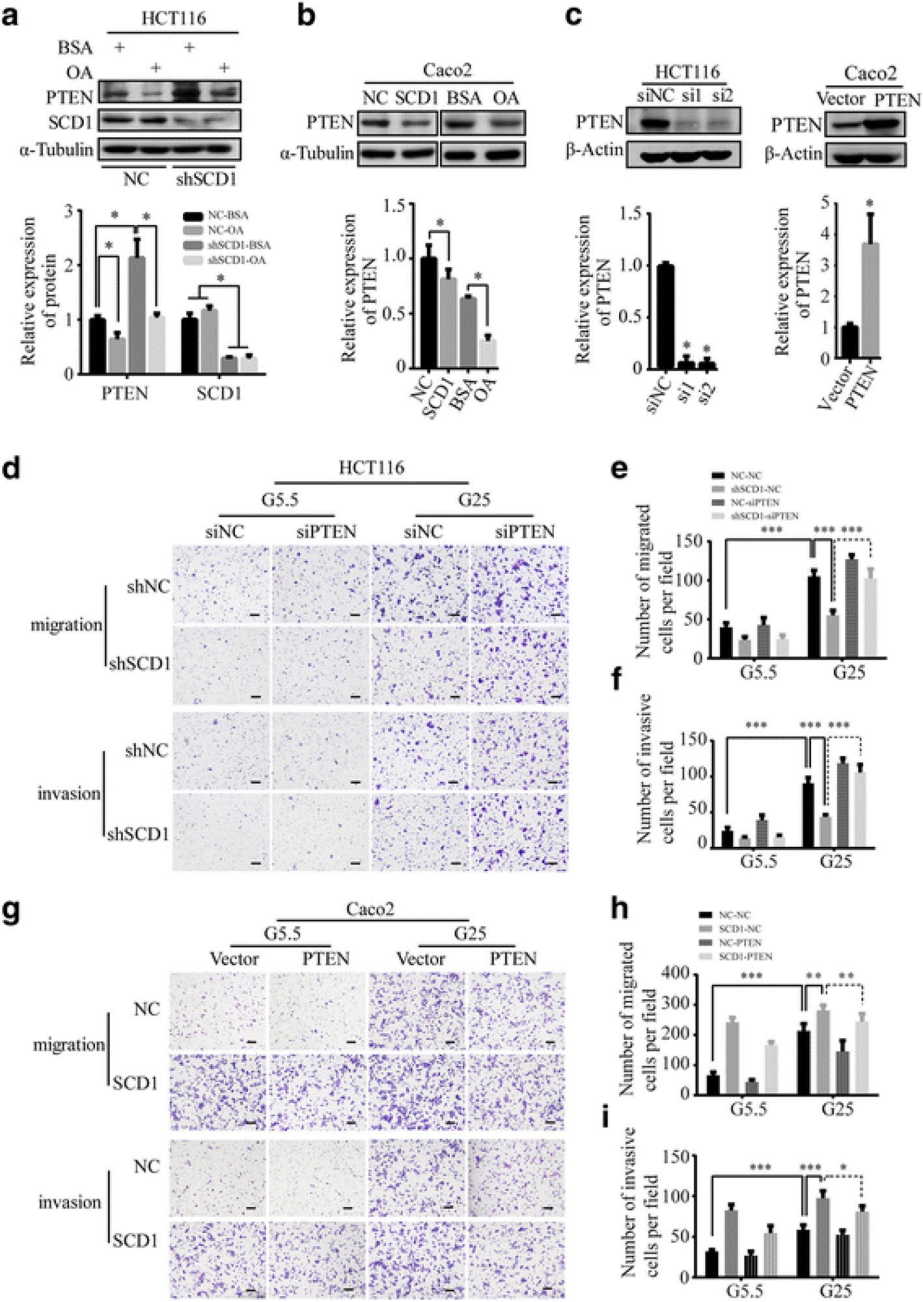
PTEN plays an important role in SCD1-induced migration and invasion of CRC cells. **a, b** Representative Western blot and quantification data of PTEN proteins of OA-treated HCT116 (**a**) and Caco2 (**b**) cells. **c** Representative Western blot and quantification data of PTEN in HCT116 cells transfected with siRNAs for PTEN (si1 and si2) or Caco2 cells ectopically expressing PTEN. **d** Representative photographs of transwell assays of shSCD1 or shNC-transfected HCT116 after being transfected with PTEN siRNAs (siPTEN) or negative control scramble siRNAs (siNC). The scale bar is 100 μm. **e**, f Histograms show the numbers of migrated (**e**) and invasive (**f**) HCT116 cells. **g** Transwell assay of SCD1 overexpression Caco2 cell lines after ectopically expressing PTEN. The scale bar is 100 μm. **h, i** Histograms show the number of migrated (**h**) and invasive (**i**) Caco2 cells

**Correct Fig. 7**

**Fig. 7:**
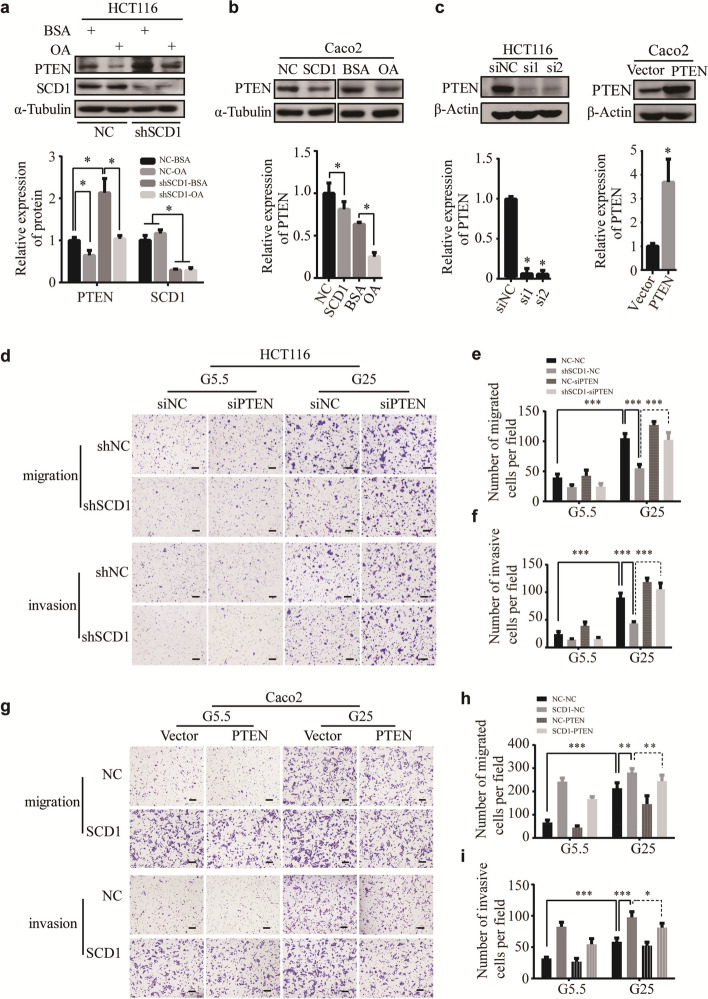
PTEN plays an important role in SCD1-induced migration and invasion of CRC cells. **a, b** Representative Western blot and quantification data of PTEN proteins of OA-treated HCT116 (**a**) and Caco2 (**b**) cells. **c** Representative Western blot and quantification data of PTEN in HCT116 cells transfected with siRNAs for PTEN (si1 and si2) or Caco2 cells ectopically expressing PTEN. **d** Representative photographs of transwell assays of shSCD1 or shNC-transfected HCT116 after being transfected with PTEN siRNAs (siPTEN) or negative control scramble siRNAs (siNC). The scale bar is 100 μm. **e**, f Histograms show the numbers of migrated (**e**) and invasive (**f**) HCT116 cells. **g** Transwell assay of SCD1 overexpression Caco2 cell lines after ectopically expressing PTEN. The scale bar is 100 μm. **h, i** Histograms show the number of migrated (**h**) and invasive (**i**) Caco2 cells

**Incorrect Figure S3**


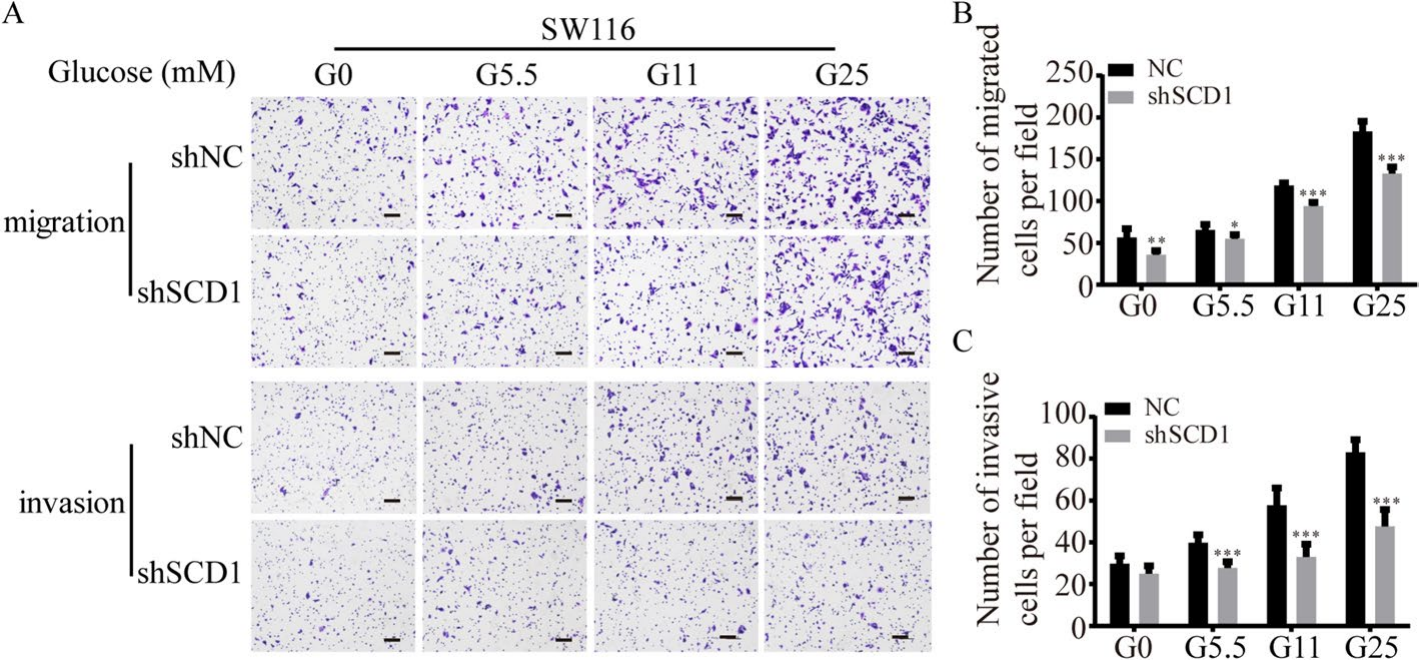
**Additional file 3: Figure S3** Effect of glucose on SCD1-induced migration and invasion ability of SW116 cells. (A) Representative photographs of transwell assays of shSCD1 or shNC-transfected SW116 cells after glucose treatment. The scale bar is 100 μm. (B, C) Histograms show the numbers of migrated (B) and invasive (C) SW116 cells. (TIFF 1277 kb)

**Correct Figure S3**


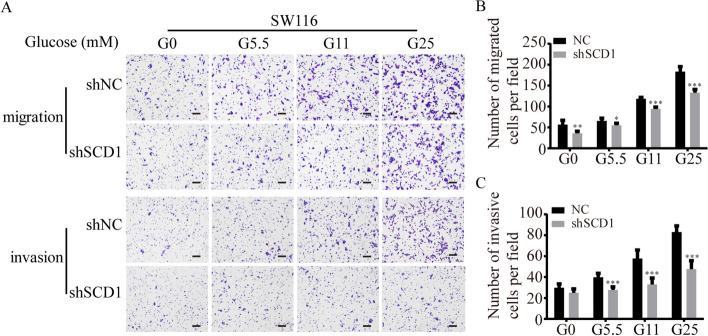
**Additional file 3: Figure S3** Effect of glucose on SCD1-induced migration and invasion ability of SW116 cells. (A) Representative photographs of transwell assays of shSCD1 or shNC-transfected SW116 cells after glucose treatment. The scale bar is 100 μm. (B, C) Histograms show the numbers of migrated (B) and invasive (C) SW116 cells. (TIFF 1277 kb)

The corrections do not compromise the validity of the conclusions and the overall content of the article.
