# Supplementary figures and images for: Stearoyl-CoA desaturase-1 promotes colorectal cancer metastasis in response to glucose by suppressing PTEN
Source: J Exp Clin Cancer Res. 2018 Mar 12;37:54. doi: 10.1186/s13046-018-0711-9 (PMC5848567; doi:10.1186/s13046-018-0711-9)

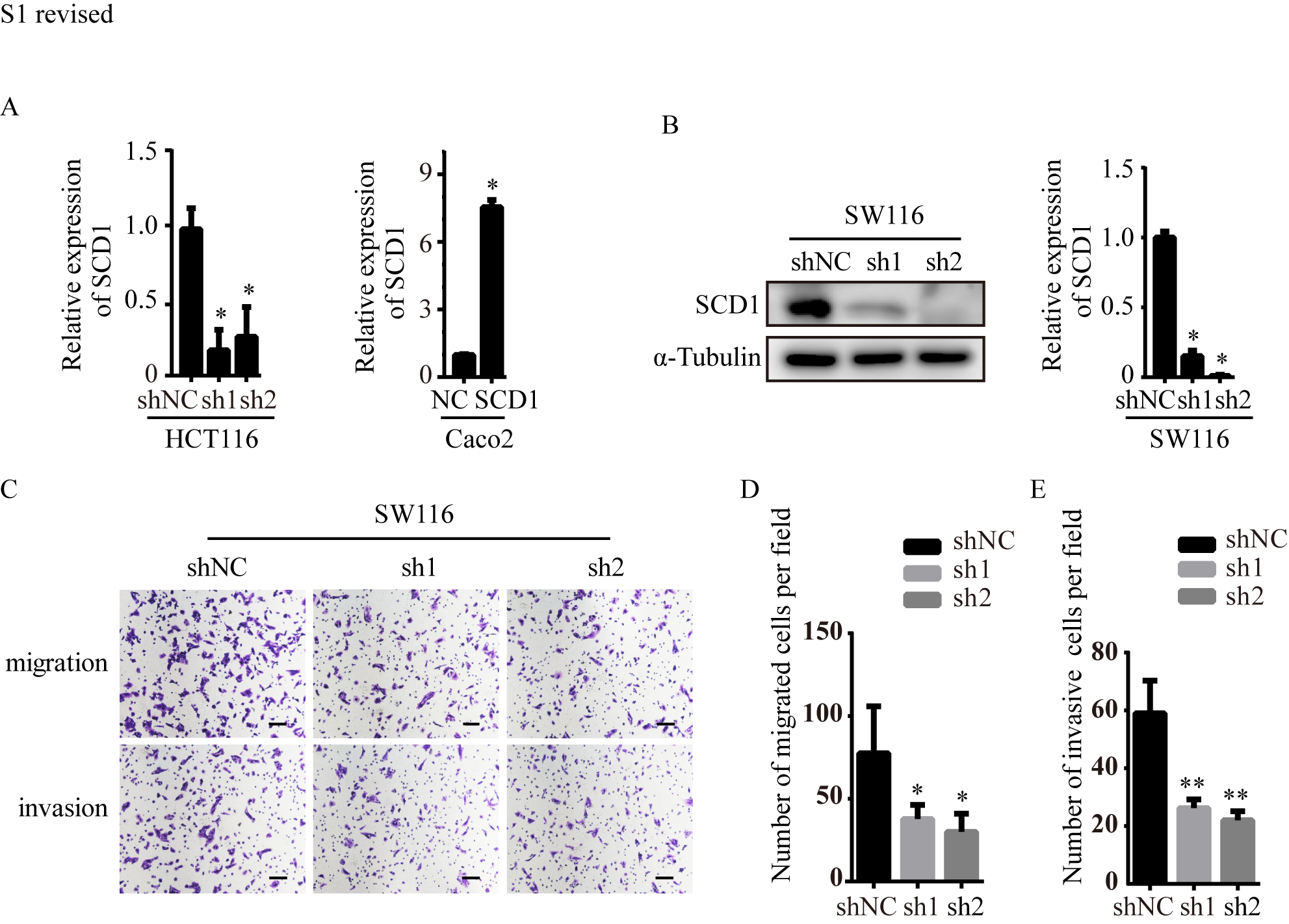

Supplement: Supplementary file 1 — Figure S1. SCD1 promotes migration and invasion of SW116 cells. (A) Quantification results of SCD1 in HCT116 cells transfected with shRNAs for SCD1 (sh1 and sh2) or Caco2 cells ectopically expressing SCD1. (B) Representative Western blot and quantified results of SCD1 in SW116 cells transfected with shRNAs for SCD1 (sh1 and sh2). (C) Transwell assay of SW116 cells after SCD1 knockdown. The scale bar is 100 μm. (D, E) Histograms show the numbers of migrated (D) and invasive (E) SW116 cells. (TIFF 1383 kb) [file 13046_2018_711_MOESM1_ESM.tif]

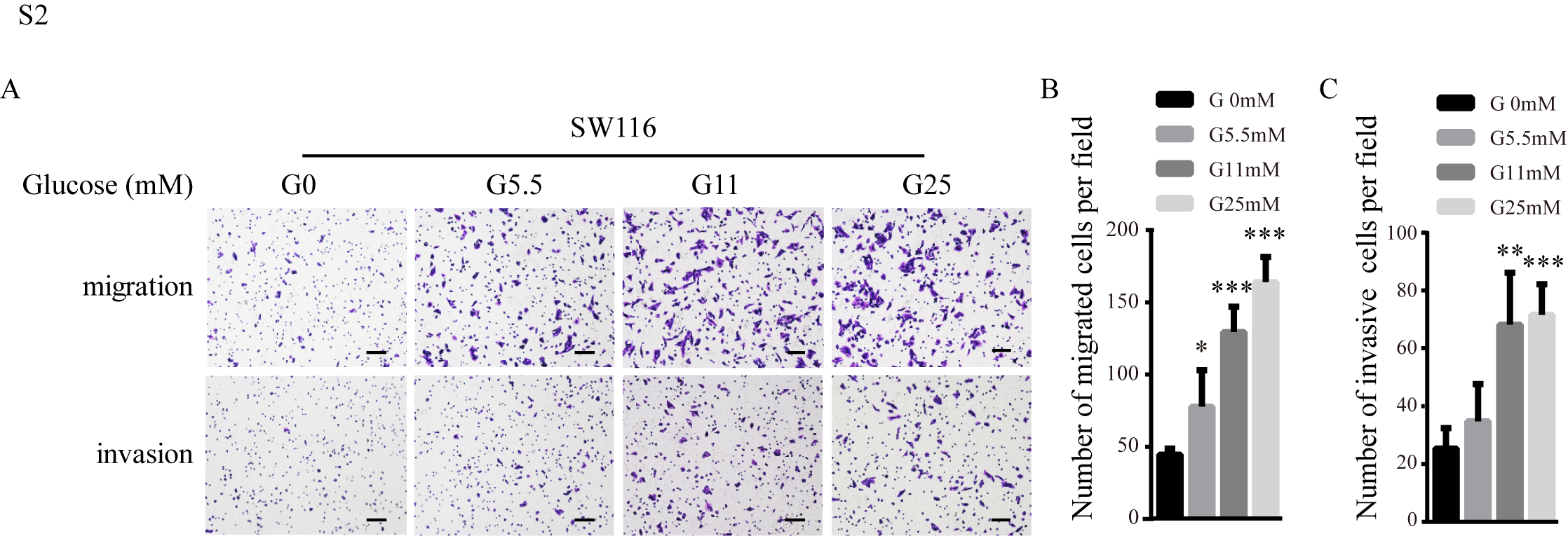

Supplement: Supplementary file 2 — Figure S2. Glucose promotes migration and invasion of SW116 cells. (A) Transwell assay of SW116 cells treated with 0 mM (G0), 5.5 mM (G5.5), 11 mM (G11) or 25 mM (G25) glucose. The scale bar is 100 μm. (B, C) Histograms show the number of migrated (B) and invasive (C) SW116 cells. (TIFF 1154 kb) [file 13046_2018_711_MOESM2_ESM.tif]

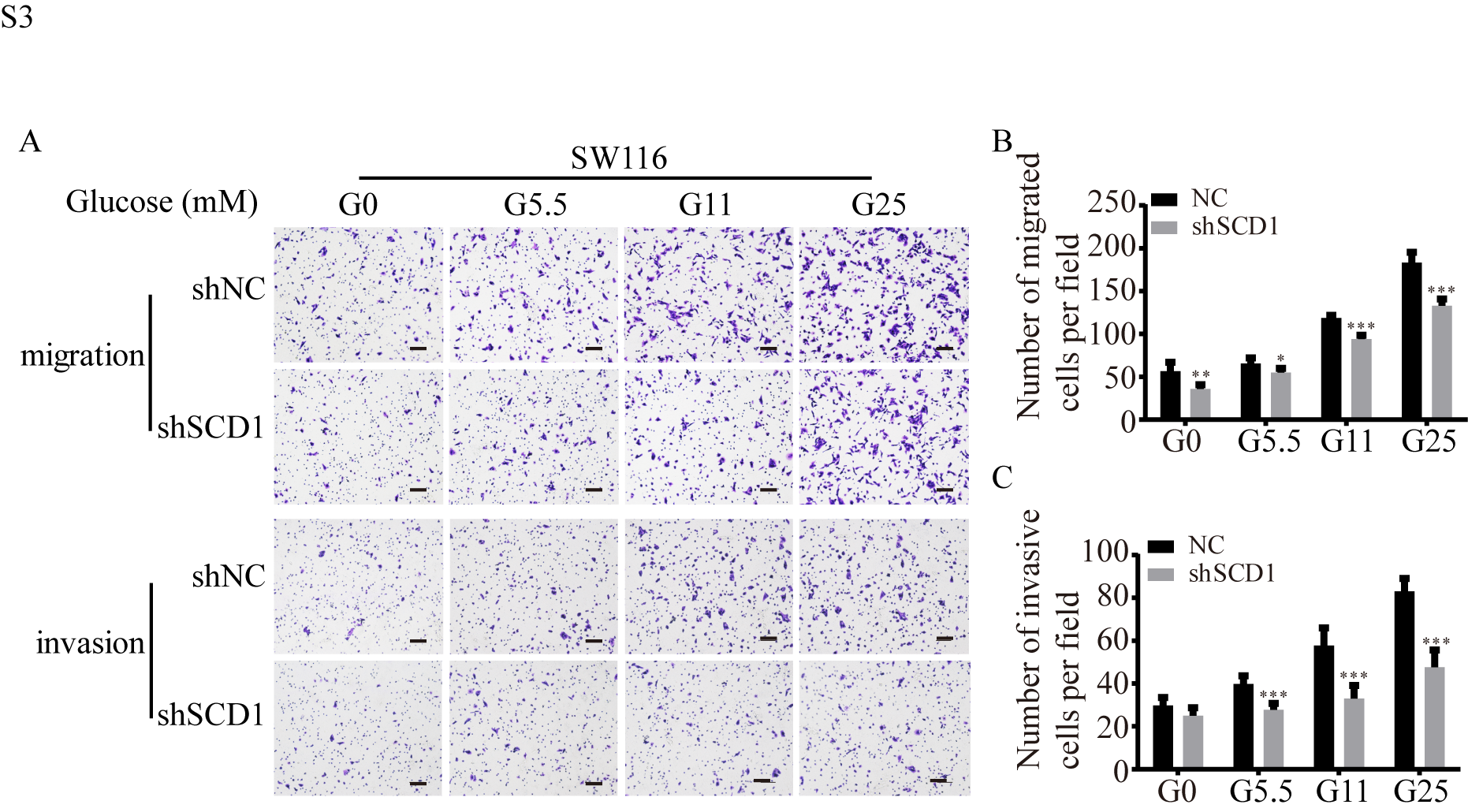

Supplement: Supplementary file 3 — Figure S3. Effect of glucose on SCD1-induced migration and invasion ability of SW116 cells. (A) Representative photographs of transwell assays of shSCD1 or shNC-transfected SW116 cells after glucose treatment. The scale bar is 100 μm. (B, C) Histograms show the numbers of migrated (B) and invasive (C) SW116 cells. (TIFF 1277 kb) [file 13046_2018_711_MOESM3_ESM.tif]

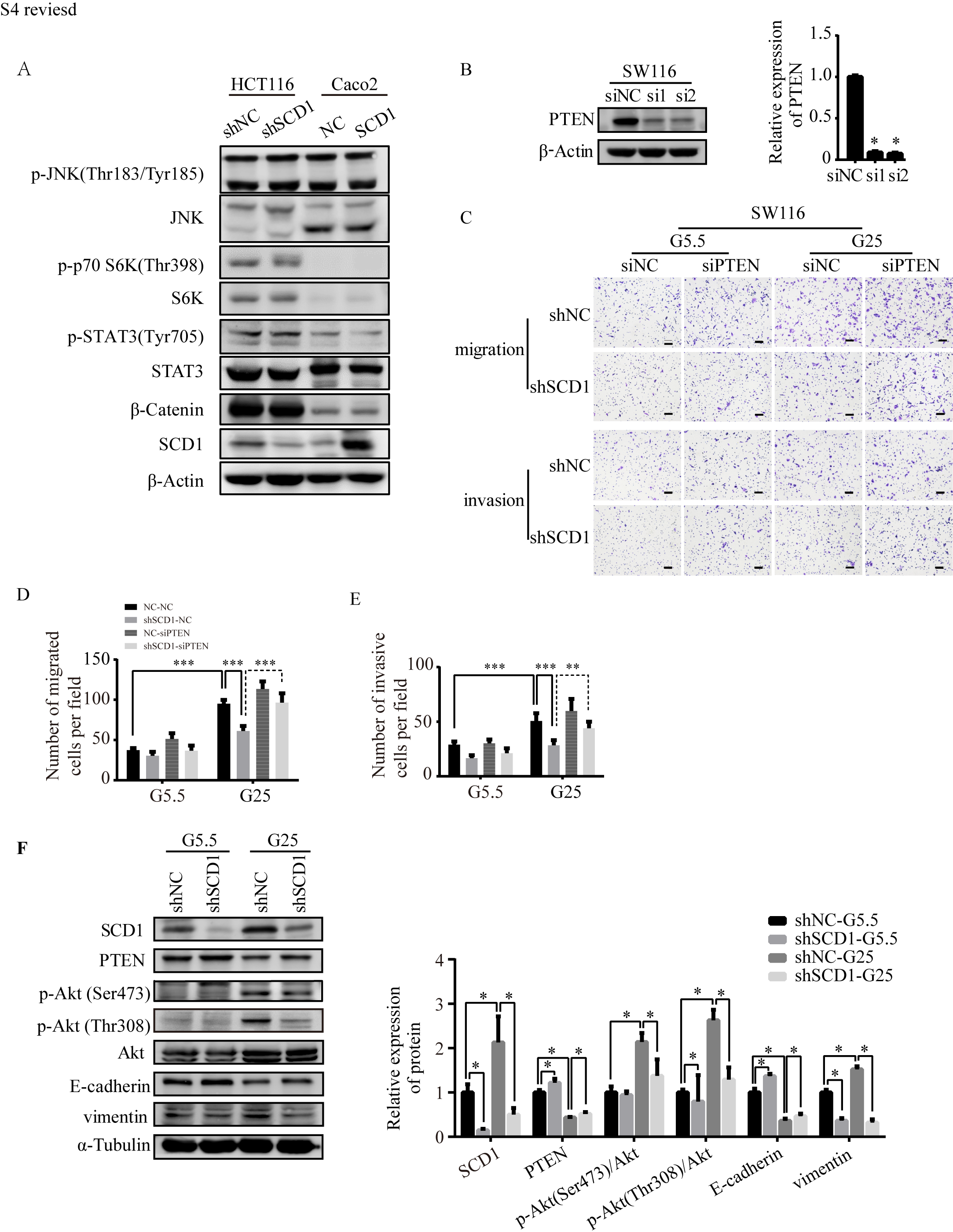

Supplement: Supplementary file 4 — Figure S4. PTEN mediates SCD1-induced migration and invasion of SW116 cells. (A) Representative Western blot of SCD1, β-Catenin, STAT3, S6K and JNK in CRC cells transfected with shSCD1 or SCD1 cDNA. (B) Representative Western blot and quantification data of PTEN in SW116 cells transfected with siRNAs for PTEN (si1 and si2). (C) Representative photographs of transwell assays of shSCD1 or shNC-transfected SW116 after being transfected with PTEN siRNAs (siPTEN) or negative control scramble siRNAs (siNC). The scale bar is 100 μm. (D, E) Histograms show the numbers of migrated (D) and invasive (E) SW116 cells. (F) Representative Western blots and quantified results of SCD1, PTEN, Akt, p-Akt (Ser473), p-Akt (Thr308), E-cadherin and vimentin. (TIFF 2175 kb) [file 13046_2018_711_MOESM4_ESM.tif]
